# Endostar, a Modified Recombinant Human Endostatin, Suppresses Angiogenesis through Inhibition of Wnt/β-Catenin Signaling Pathway

**DOI:** 10.1371/journal.pone.0107463

**Published:** 2014-09-18

**Authors:** Xiaoming Xu, Wei Mao, Qian Chen, Qin Zhuang, Lihui Wang, Jin Dai, Haibing Wang, Zhaoquan Huang

**Affiliations:** 1 Department of Cardiology, Zhejiang Provincial Hospital of Traditional Chinese Medicine, Hangzhou, Zhejiang, China; 2 First College of Clinical Medicine, Zhejiang Chinese Medical University, Hangzhou, Zhejiang, China; University of Regensburg, Germany

## Abstract

Endostar, a novel modified recombinant human endostatin, is now widely studied for the treatment of diseases that are characterized or caused by pathological angiogenesis. However, its molecular mechanism remains unclear. In this study, we investigated the effects of Endostar on the Wnt/β-catenin signaling pathway, which has emerged as an important aspect of angiogenesis. We showed that Endostar significantly inhibited the proliferation, migration, invasion, and capillary-like tube formation of human umbilical vascular endothelial cells in a dose-dependent manner. Using a luciferase reporter assay, we also demonstrated that Endostar suppressed β-catenin-dependent T cell factor transcriptional activity in increasing doses. Moreover, we found that Endostar treatment also restricted the stabilized mutant β-catenin-mediated increase in transcriptional activity, suggesting that Endostar exerts its inhibitory influence on Wnt/β-catenin signaling by targeting β-catenin or its downstream molecules. Western blot and immunofluorescence results revealed that Endostar significantly decreased nuclear and total β-catenin levels. Finally, we discovered that Endostar down-regulated cyclin D1 and VEGF, two proteins that are known as the downstream targets of Wnt/β-catenin signaling and that also play important roles in angiogenesis. Our findings suggested that Endostar inhibits angiogenesis and that the downregulation of the Wnt/β-catenin signaling pathway may be involved in the inhibition of angiogenesis by Endostar. These results support the use of Endostar in further clinical applications.

## Introduction

Angiogenesis, or the formation of new capillaries from preexisting blood vessels, is involved in the pathogenesis of many diseases such as cancer, atherosclerosis, and diabetic retinopathy [1]–[3]. A growing body of evidence shows that anti-angiogenesis therapy may become one important approach to the treatment of these diseases [4]–[6].

Endostatin, a 20 kD C-terminal fragment of collagen XVIII, is one of the most effective anti-angiogenesis agents available. It has already been used to treat atherosclerosis and was also pushed into anti-cancer clinical trials [7], [8]. However, the agent’s instability diminished its efficacy in those studies. A new recombinant human endostatin with an additional nine amino acid (MGGSHHHHH) sequence at the N terminus of its protein, Endostar, was more stable and was shown to be at least twice as potent as endostatin in animal tumor models by Folkman [9]. In 2005, the State Food and Drug Administration of China approved the use of Endostar for the treatment of non-small-cell lung cancer. It has also been reported that Endostar attenuates the progression of adjuvant arthritis because of its anti-angiogenesis capabilities [10]. In our previous study, we found that Endostar inhibits neovascularization and plaque growth in the rabbit atherosclerosis model [11].

Despite extensive studies of Endostar’s effects on these diseases, its molecular mechanism still remains ambiguous. Previous research showed that Endostar restricts angiogenesis by blocking VEGF-induced tyrosine phosphorylation of VEGFR-2 and inducing apoptosis through the activation of caspase-3 and decrease of Bcl-2 in human umbilical vein endothelial cells (HUVECs) [12], [13]. Endostar also down-regulates hypoxia-inducible factor 1 and VEGF expression in human lung adenocarcinoma cancer cells [14].

Recent studies emphasize the important role of the Wnt/β-catenin signaling pathway in physiological and pathological angiogenesis [15], [16]. The Wnt/β-catenin pathway is activated when a Wnt ligand binds to its coreceptor complex, which contains a Frizzled family member and its low-density lipoprotein receptor-related protein 5/6 (LRP-5/6) [17], [18]. Wnt signaling then induces Dishevelled phosphorylation and results in the dissociation of the destruction complex, which includes axin, adenomatous polyposis coli, and glycogen synthase kinase 3β (GSK-3β), and the stabilization of β-catenin [17], [18]. Cytoplasmic β-catenin accumulates and travels into the nucleus, then forms complexes with the T cell factor/lymphoid enhancer binding factor (TCF/LEF) family [17], [18]. Finally, the complexes stimulates transcription of Wnt target genes including cyclin D1, interleukin-8, and lymphoid enhancer-binding factor 1, which regulates the fundamental aspects of angiogenesis including cellular polarity, proliferation, survival, and branching morphogenesis [17]–[21].

In this study, we assessed the anti-angiogenic efficacy of Endostar in vitro and investigated its mechanism of action on the Wnt/β-catenin pathway in HUVECs.

## Materials and Methods

### Reagents

Endostar, expressed and purified in E.coli, was purchased from Simcere Pharmaceutical Research Co., Ltd. (Shandong, China). Primary antibodies for β-catenin, cyclin D1, and β-actin were obtained from Cell Signaling Technology (MA, USA). Primary antibodies for VEGF and Histone H3 were obtained from Novus Biologicals (MA, USA) and Epitomics (CO, USA). pGL3-OT or pGL3-OF luciferase reporter gene plasmid and pcDNA3.1+ empty plasmid were kindly provided by Dr. Dai SD (China Medical University, Shenyang, China). The pcDNA3-S33Y-β-catenin plasmid and pRL-SV40 plasmid were obtained from Addgene (Plasmid 19286) and Promega (Madison, WI, USA), respectively.

### Cell culture

HUVECs were purchased from Sciencell (Carlsbad, CA, USA) and were cultured in endothelial cell medium (ECM; Sciencell, Carlsbad, CA, USA) which contained 5% fetal bovine serum (FBS), 1% endothelial cell growth supplement (ECGS), 100 U/ml penicillin, and 100 U/ml streptomycin at 37°C under a humidified 95%: 5% (v/v) mixture of air and CO2. The HUVECs were collected for use after 3–5 passages.

### Cell viability assay

As described previously, an MTT (Amresco, USA) assay was used to determine cell viability [22]. HUVECs were seeded into 96-well plates at 1×10^4^ cells/well for 12 hours, then incubated with different concentrations of Endostar (50, 100 and 150 µg/ml) for 24 hours. HUVECs without Endostar treatment was used as a control. Cell viability was determined by the MTT method and calculated as % of control.

### Wound-healing migration assay

We used a wound healing assay to determine the chemotactic motility of HUVECs. The HUVECs were grown into full confluence in 6-well plates and starved with ECM containing 0.5% FBS for 6 hours to deactivate cell proliferation. After that, we wounded the HUVECs with pipette tips and washed them with PBS. We then incubated the cells in ECM with 5% FBS, 1% ECGS and different concentrations of Endostar (50, 100 and 150 µg/ml) for 12 hours. We took images of the HUVECs and manually counted the ones that had migrated into the cell’s free space.

### Transwell invasion assay

We performed an invasion assay using the Transwell (Corning, MA, USA) with 6.5-mm diameter polycarbonate filters (8 µm pore size). Briefly, the Transwell was coated with 25 µg growth factor reduced Matrigel (BD Biosciences, MA, USA) and incubated for 2 hours at 37°C for gelling. The bottom chambers were filled with 500 µl ECM with 5% FBS, 1% ECGS to act as a chemoattractant [22] and the top chambers contained seeded starved HUVECs (4×10^4^) in 100 µl ECM (0.5% FBS) and different concentrations of Endostar (50, 100 and 150 µg/ml) for 12 hours. Noninvasive cells on the upper surface of the filter were then removed with cotton swabs. The Transwell inserts were fixed with cold 4% paraformaldehyde, stained with 0.1% crystal violet for 15 minutes, and washed with PBS for three times. Afterward, the filter was mounted onto glass slides and the invasive cells on the lower side of the filter were counted under 100× magnification from three random fields.

### Tube formation assay

We used a protocol from BD Biosciences to assess tube formation. Briefly, we coated pre-chilled 24-well plates with 289 µl growth factor reduced Matrigel and incubated them at 37°C for 45 minutes. Inactivated HUVECs (4×10^5^/ml) were suspended in ECM (5% FBS, 1% ECGS) with different concentrations of Endostar (50, 100 and 150 µg/ml), and 300 µl of the cell suspension was added to each well. After 16 hours, we photographed the tubular structure of endothelial cells with an inverted microscope (Nikon, Japan) at 100× magnification. We then counted branch points in three random view-fields per well.

### Dual-luciferase assay

The TOPFlash/FOPFlash luciferase assay is widely used to evaluate β-catenin/TCF dependent signaling events that regulated by TCF/LEF family. TOPflash (pGL3-OT) construct driven by thymidine kinase minimal promoter contains three wild-type TCF binding sites upstream of a luciferase reporter gene. FOPflash (pGL3-OF) contains three mutated TCF binding sites and is a specificity control for TOPflash activity [23]. We evaluated the effects of Endostar on TCF/LEF-dependent transcriptional activity in HUVECs by performing a luciferase reporter assay with pGL3-OT and pGL3-OF. We transfected 10^5^ cells that were grown in 24-well culture plates with 200 ng of pGL3-OT or pGL3-OF reporter gene plasmids, and 10 ng of pRL-SV40 using Lipofectamine LTX/Plus Reagent (Invitrogen, USA). For our cotransfection experiments, we transfected cells with 200 ng of pcDNA3-S33Y-β-catenin plasmid or a control empty plasmid plus the reporter plasmid. After the cells were incubated for 12 hours, we added different concentrations of Endostar (50 and 100 µg/ml) into each well. After 24 hours, we used the Dual Luciferase Reporter Assay System (Promega USA) and a luminometer (Bio-Rad, USA) to measure the luciferase activities. TCF/LEF-dependent transcriptional activity was normalized to Renilla luciferase activity from the control plasmid pRL-SV40.

### Western blotting

We further analyzed the effects of Endostar on Wnt/β-catenin signaling pathway. HUVECs were incubated in ECM (5% FBS, 1% ECGS) with different concentrations of Endostar (50, 100 and 150 µg/ml) for 24 hours. The whole-cell extracts and the nuclear extracts were each prepared using RIPA buffer (Beyotime, China) and NE-PER nuclear and cytoplasmic extraction reagents (Thermo Scientific, USA) respectively. Proteins were separated by polyacrylamide gel electrophoresis and transferred to polyvinylidene difluoride (PVDF) membranes (Bio-Rad, USA). The membranes were incubated with primary antibodies anti-β-catenin, anti-VEGF, anti-cyclin D1, anti-β-actin and anti-Histone H3 followed by the addition of secondary antibody (Goat anti-rabbit or mouse IgG (H+L) antibody, KPL, USA). Protein bands were detected with an enhanced chemiluminescent substrate (EZ-ECL, Biological Industries, Kibbutz Beit-Haemek, Israel), scanned, and quantitated by a Bio-imaging analyzer (Bio-Rad).

### Immunofluorescence

We analyzed the effects of Endostar on the cellular localization of β-catenin. HUVECs were grown overnight in confocal laser dishes and then treated with or without 20 mmol/l LiCl (Amresco, USA), a dose known to activate β-catenin [24], [25], in the presence or absence of 100 µg/ml Endostar. After 24 hours, the cells were fixed in 4% paraformaldehyde in PBS for 10 minutes, and punched with 0.5% Triton for 15 minutes. The cells were then incubated with anti-β-catenin antibody overnight, followed by the Cy3 conjugated Goat anti-Rabbit IgG (H+L) antibody (1∶500, Jackson ImmunoResearch, USA) for 1 hour at room temperature. Cellular nuclei were labeled with the fluorescent dye DAPI at 1∶5000 dilutions for 1 hour. Images were photographed with a confocal microscope (ULTRAVIEW Vox, PE).

### Statistical analysis

All experiments were performed in duplicate and repeated at least three times. Quantitative data were presented as mean ± standard deviation (SD). Data comparison was based on analysis of variance (ANOVA), followed by a Bonferroni test to establish differences among groups (SPSS 17.0). Significant differences were accepted when p<0.05.

## Results

### Endostar inhibits the proliferation of HUVECs

**Figure 1:**
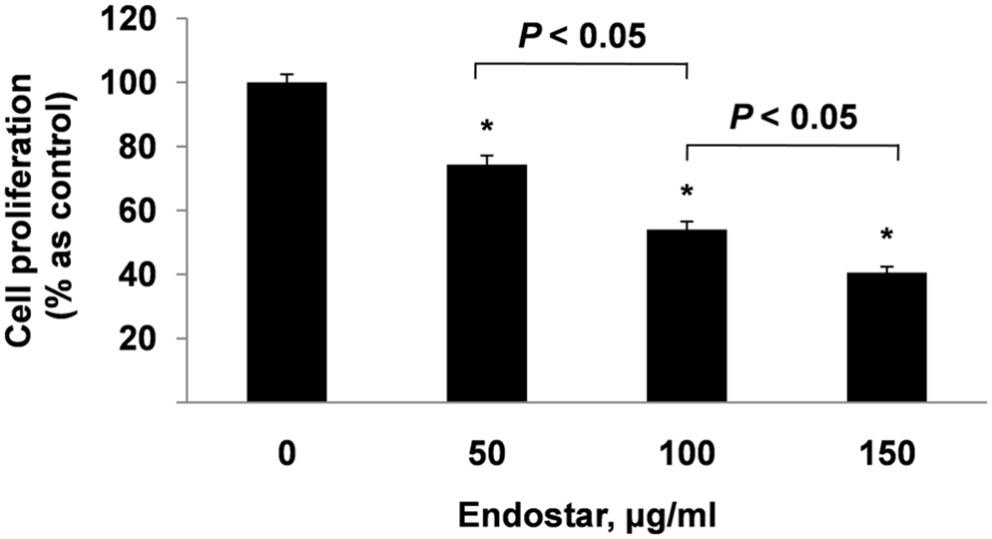
Endostar inhibits the proliferation of HUVECs in a dose-dependent manner. HUVECs (1×10^4^/well) were incubated in ECM (5% FBS, 1% ECGS) with different concentrations of Endostar for 24 h. Cell viability was quantified by an MTT assay. *P<0.05 versus the control group.

An MTT assay showed that the proliferation of HUVECs in Endostar-treated groups (50, 100, and 150 µg/ml) was suppressed by 25.63±2.85%, 45.92±2.50%, and 59.47±1.86%, respectively, after 24 hours (Figure 1). Therefore, the cell proliferation of HUVECs was inhibited in a dose-dependent manner by treatment with Endostar.

### Endostar inhibits the migration of HUVECs

**Figure 2:**
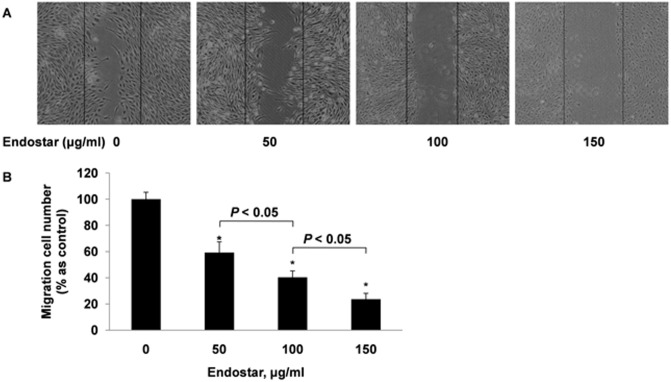
Endostar inhibits HUVECs migration in a dose-dependent manner. Cell migration was measured by wound-healing migration assay. HUVECs were treated with different concentrations of Endostar for 12 h and the migrated cells were quantified by manual counting. *P<0.05 versus the control group.

We performed a wound healing assay to measure the effects of Endostar on HUVEC migration. As shown in Figure 2, Endostar significantly restricted the migration of HUVECs by 40.72±8.10%, 59.62±4.90% and 76.29±4.40%, respectively. The inhibition at 150 µg/ml of Endostar was very similar to the suppression observed at a zero hour incubation (Figure 2).

### Endostar inhibits the invasion of HUVECs

**Figure 3:**
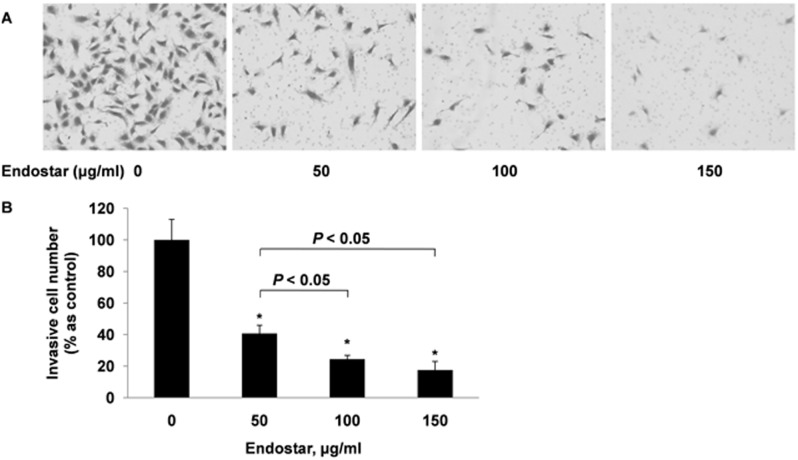
Endostar inhibits HUVECs invasion in Transwell invasion assay. HUVECs were seeded in the top chamber of Transwell and treated with different concentrations of Endostar. The bottom chambers were filled with 500 µl ECM with 5% FBS and 1% ECGS. After 12 h, the invasive HUVECs were stained and quantified by manual counting. *P<0.05 versus the control group.

Transwell assays were used to further assess Endostar’s effects on the chemotactic motility of HUVECs. Only a few invasive cells were observed in the 150 µg/mL Endostar-treated groups (Figure 3). Therefore, we found that 50, 100, and 150 µg/ml of Endostar significantly inhibited the invasion of HUVECs by 59.31±5.21%, 75.52±2.39% and 82.41±5.38%, respectively, when compared with the control (Figure 3).

### Endostar restricts the tube formation of HUVECs

**Figure 4:**
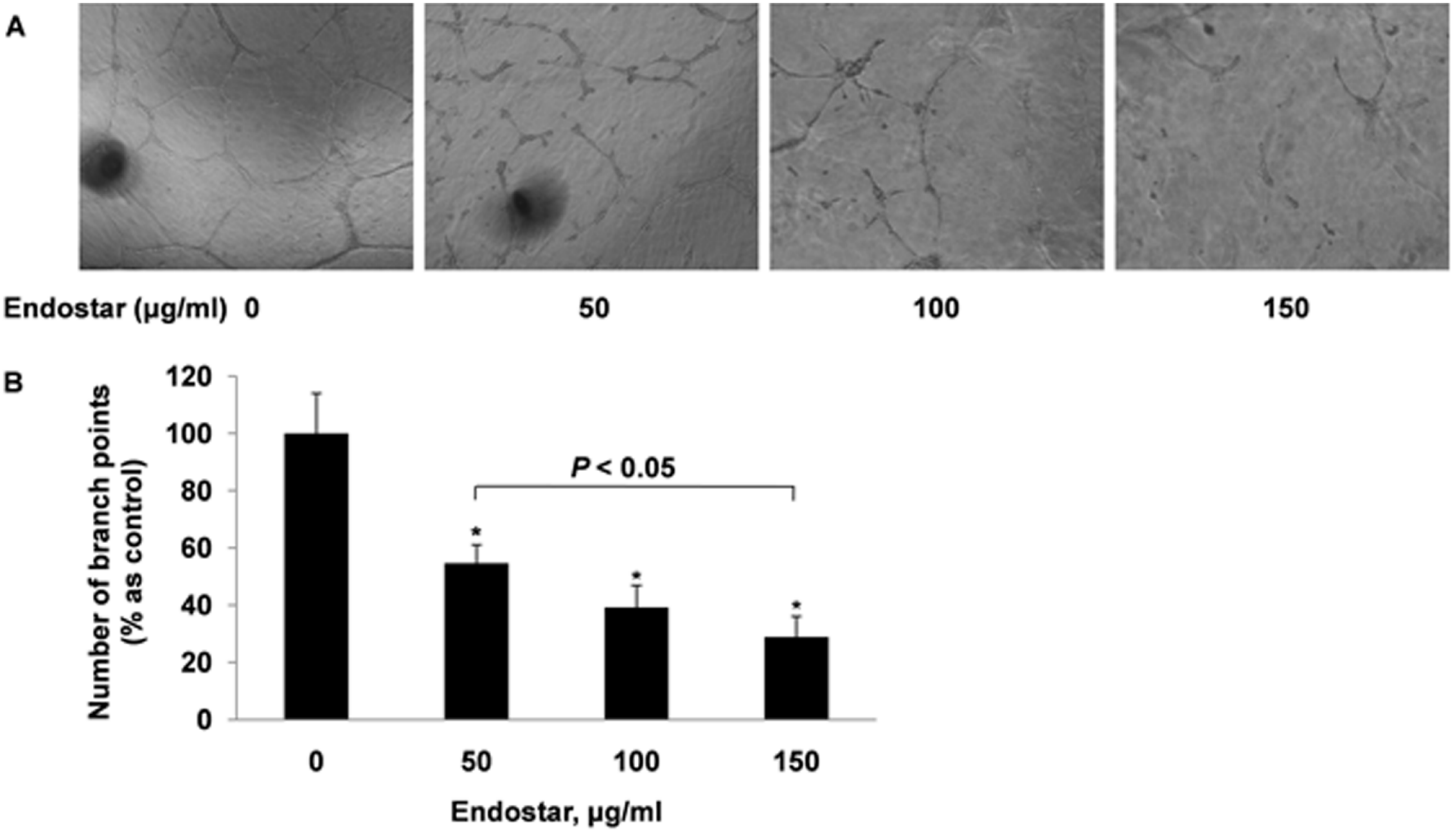
Endostar inhibits the tube formation HUVECs. HUVECs were seeded on 24-well Matrigel coated plates at a density of 1.2×10^5^ cells/well. After treated with different concentrations of Endostar for 16 hours, capillary tube structures were photographed (magnification×100) and the branch points in three random fields per well were quantified. Representative capillary tube structures were shown. *P<0.05 versus the control group.

Endothelial cell tube formation is an essential step in angiogenesis [26]. Therefore, we evaluated the effects of Endostar on HUVEC angiogenesis with a tube formation assay. HUVECs were seeded on the growth factor reduced Matrigel, and capillary tube structures formed in the control group at 16 hours (Figure 4). However, Endostar significantly reduced tubular formation of endothelial cells at 50, 100 and 150 µg/ml by 45.36±6.44%, 60.82±7.78% and 71.13±7.14%, respectively (Figure 4).

### Endostar inhibits Wnt/β-catenin signaling pathway in HUVECs

**Figure 5:**
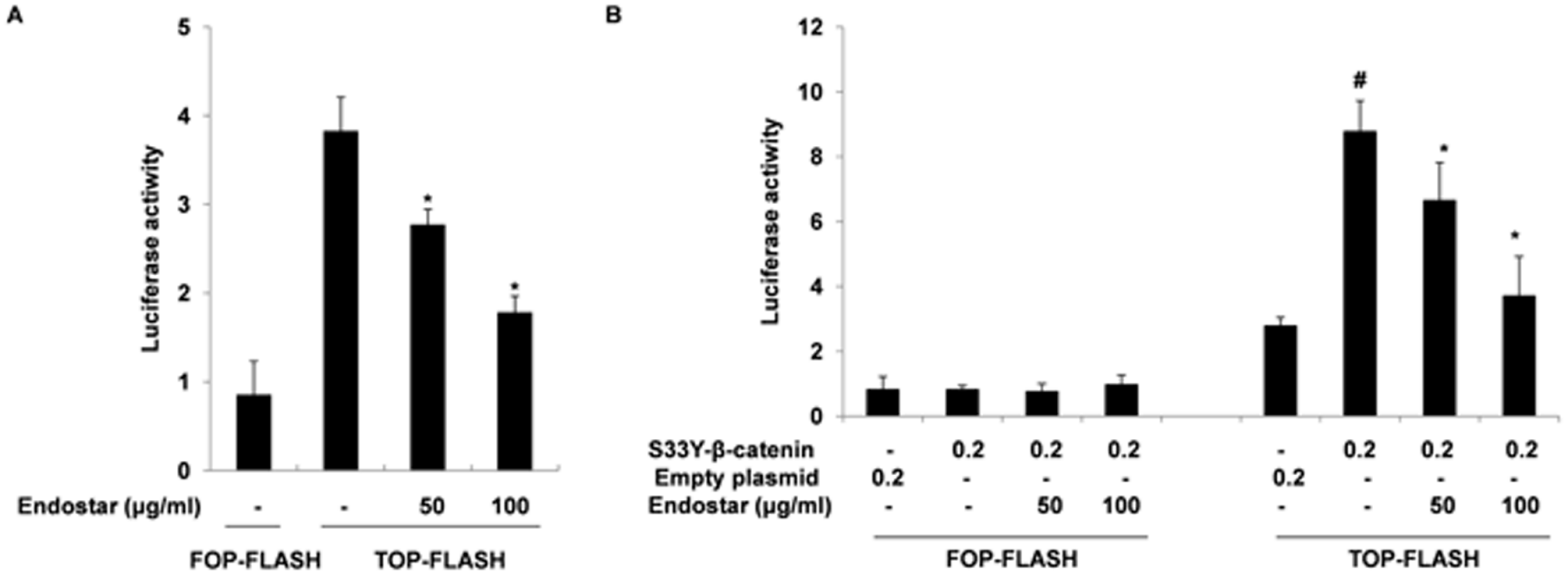
Endostar inhibits TCF/LEF-dependent transcriptional activity in HUVECs. (A) Inhibition of TCF/LEF-dependent transcriptional activity in HUVECs by Endostar. HUVECs were transfected with pGL3-OT (a TCF/LEF-responsive reporter) or pGL3-OF (a negative control) plasmids plus pRL-SV40 as internal control and treated with different concentrations of Endostar. After 24 h, the luciferase activities were measured using the dual-luciferase reporter assay system and normalized to Renilla luciferase activity. *P<0.05 versus the control group. (B) Endostar suppresses the mutant β-catenin-mediated increase in TCF/LEF-dependent transcriptional activity. The pcDNA3-S33Y-β-catenin (a constitutively stabilized mutant β-catenin) plasmid and the reporter plasmid were cotransfected into HUVECs. After 12 h, cells were incubated with different doses of Endostar and the luciferase activities were determined.^ #^P<0.05 versus the empty vector group. *P<0.05 versus the S33Y-β-catenin group.

Angiogenesis is a very complex process that is mediated by many signaling routes, including the Wnt/β-catenin signaling pathway [15], [27]. To investigate whether Endostar could suppress Wnt/β-catenin signaling pathway in HUVECs, we first examined influences of Endostar on the TCF/LEF-dependent transcriptional activity using a dual-luciferase reporter assay. At 24 hours, 50 and 100 µg/ml Endostar inhibited TCF/LEF-dependent transcriptional activity in HUVECs by 27.68±4.44% and 53.52±4.96%, respectively (Figure 5A). Since β-catenin is an essential mediator of Wnt/β-catenin pathway, we next cotransfected the HUVECs with pcDNA3-S33Y-β-catenin (a constitutively stabilized mutant β-catenin) plasmid plus the reporter plasmid. As shown in Figure 5B, the TCF/LEF-dependent transcriptional activity increased by 2.15-fold, when compared with the empty vector. Endostar treatment suppressed this increased activity in a dose-dependent manner (Figure 5B). These results suggested that Endostar exerts its inhibitory influence on Wnt/β-catenin pathway by targeting β-catenin or its downstream molecules.

**Figure 6:**
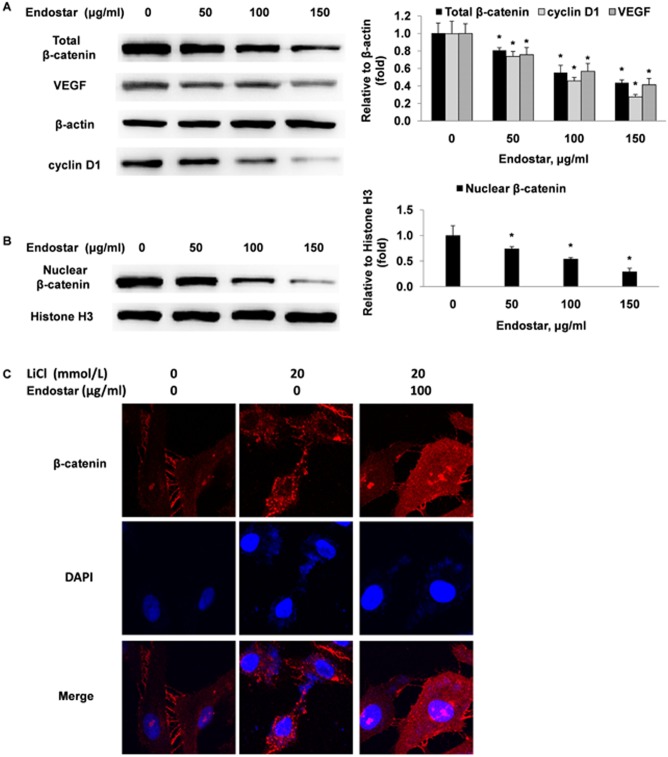
Endostar suppresses the expression of nuclear and total cellular β-catenin, cyclin D1 and VEGF in HUVECs. (A and B) HUVECs were incubated with different concentrations of Endostar for 24 h. The whole-cell extracts and the nuclear extracts were prepared and analyzed by Western blotting and probed with specific antibodies. *P<0.05 versus the control group. (C) HUVECs were treated with or without 20 mmol/l LiCl (a dose known to activate β-catenin) in the presence or absence of 100 µg/ml Endostar. After 24 h, the expression and cellular localization of β-catenin (red) was determined by immunofluorescence analysis. The nucleus was stained with DAPI (blue).

We then investigated the effects of Endostar on the expression of nuclear and total β-catenin and cyclin D1 (a downstream target gene) using a western blotting assay. The results in Figures 6A and 6B revealed that nuclear and total β-catenin levels in HUVECs were decreased by increasing doses of Endostar. Cyclin D1, a protein essential for cell proliferation, was also down-regulated by Endostar treatment (Figure 6A). Compared with the control, 100 µg/ml Endostar reduced nuclear and total β-catenin and cyclin D1 levels by 45.67±2.31%, 44.67±8.15%, and 53.33±4.04%, respectively. VEGF is one of the most important regulators of angiogenesis. In our study, we found that Endostar could dose-dependently inhibit the expression of VEGF in HUVECs (Figure 6A). We also performed immunofluorescence to detect the expression and cellular localization of β-catenin. LiCl, a potent GSK-3β inhibitor, was used to increase the β-catenin level in HUVECs. As shown in Figure 6C, the intensity of nuclear and cytoplasmic β-catenin was increased by LiCl treatment, and 100 µg/ml Endostar significantly reduced the intensity of nuclear and cytoplasmic β-catenin, as compared with its control. These results indicated that Endostar inhibits Wnt/β-catenin pathway activity by reducing the expression of nuclear and total β-catenin.

## Discussion

Pathological angiogenesis plays an important role in many diseases such as cancer, atherosclerosis, and diabetic retinopathy, and is a strategic target for future clinical therapy. Though Endostar, a novel recombinant human endostatin, is now widely studied for the treatment of these diseases, its molecular mechanism remains ambiguous. In the present report, we have shown that Endostar inhibits angiogenesis and that the downregulation of the Wnt/β-catenin signaling pathway may be involved in the inhibition of angiogenesis by Endostar.

In this study, we first assessed the anti-angiogenic effects of Endostar in vitro. Our results showed that Endostar significantly inhibited multiple steps of angiogenesis, including endothelial cell proliferation, migration, invasion, and capillary structure formation in a dose-dependent manner, which is consistent with findings from previous studies [12]. However, the concentration range of Endostar previously used is still uncertain in vitro. Yun Ling et al. found that 5–125 µg/ml Endostar significantly inhibited VEGF-mediated angiogenesis in HUVECs [12]. XingQi Li et al. treated HUVECs with 250 µg/ml Endostar, and found that VEGF-mediated angiogenesis was significantly inhibited [28]. Dandan Yu et al. reported that when Endothelial progenitor cells were treated with 50–400 µg/ml Endostar, the microvascular endothelial cell growth medium-2-stimulated angiogenesis was significantly inhibited by Endostar treatment [29]. In our present study, we found that Endostar (50–150 µg/ml) significantly inhibited ECM (5% FBS, 1% ECGS)-mediated angiogenesis in a dose-dependent manner. Fengyan Jin et al. demonstrated that Endostar (200–400 µg/ml) and oxaliplatin synergistically inhibited the colorectal cancer cell line SW1116 proliferation, Matrigel adhesion and invasion [30]. Therefore, the concentration ranges of Endostar in vitro may be closely correlated with culture conditions and cell types.

Increasing evidence indicates that the Wnt/β-catenin signaling pathway plays an important role both in physiological and pathological angiogenesis [15], [16]. Many Wnt ligands (Wnt2b, Wnt3, Wnt4, Wnt5a, etc.), Frizzled receptors (Frizzled1, Frizzled2, Frizzled6, Frizzled8, etc.), and LRP-5/6 are widely expressed by cultured endothelial cells [31]. Moreover, high activation of β-catenin transcriptional signaling is detected during embryonic development in many types of vessels including retina and brain microvasculature [32]. Also, endothelial-specific deletion of β-catenin leads to early lethality resulting from defective vascular remodeling and diffuse hemorrhages [33]. In diabetic retinopathy, neovascularization is accompanied by increased β-catenin nuclear signaling, which promotes retina vascularization [34]. Interestingly, SERPINA3 K, which acts as a Wnt signaling inhibitor by binding to LRP 6, reduced diabetic retinopathy [34].

Research that highlighted the emerging importance of the Wnt/β-catenin pathway in angiogenesis led us to assess the effects of Endostar on this pathway in HUVECs. In this study, we found that Endostar significantly inhibited TCF/LEF-dependent transcriptional activity in HUVECs in a dose-dependent manner. The phosphorylation of ser-33 is necessary for β-catenin degradation, and S33Y-β-catenin, a constitutively stabilized mutant β-catenin, is insensitive to the upstream antagonists of Wnt signaling [35]. Expression of S33Y-β-catenin heightened the TCF/LEF-dependent transcriptional activity 2.15-fold. Yet Endostar treatment suppressed this increased action, indicating that the endostatin restricts Wnt/β-catenin pathway activity by targeting β-catenin or its downstream molecules. Consequently, we examined whether Endostar affected the nuclear and total β-catenin expression in HUVECs. We discovered that nuclear and total β-catenin levels were significantly decreased by Endostar in a dose-dependent manner. Previous studies have shown that LiCl can inhibit GSK-3β and subsequently increase the β-catenin level in HUVECs [25]. Using immunofluorescence analysis, we also found that, compared with the control, Endostar reduces the intensity of nuclear and cytoplasmic β-catenin. The Wnt/β-catenin signaling pathway affects the expression of many genes relevant to angiogenesis, such as cyclin D1, VEGF, c-myc, interleukin-8 and lymphoid enhancer-binding factor 1, etc. [17]–[21]. Cyclin D1 plays an important role in G0/G1 to S transition, resulting in increased cell proliferation [36], [37]. Previous study showed that Endostar induced G0/G1 phase cell cycle arrest in endothelial cells [38]. It is also demonstrated that cyclin D1 promotes cellular migration through thrombospondin 1 and Rho-activated kinase II [39]. Therefore, we further learned that Endostar treatment down-regulates cyclin D1 in a dose-dependent manner. Our results suggested that Endostar suppressed Wnt/β-catenin pathway activity by reducing the expression of nuclear and total β-catenin.

VEGF is one of the most important regulators of angiogenesis [40], [41]. In our study, we found that treatment with Endostar resulted in a significant decrease of VEGF expression in HUVECs. Previous studies have shown that activation of Wnt/β-catenin signaling can upregulate VEGF expression in colon cancer cells and in human endothelial cells [42]–[44]. Therefore, it is conceivable that the inhibitory effect of Endostar on VEGF expression may partly contribute to the suppression of the Wnt/β-catenin signaling pathway.

## Conclusions

In summary, our study shows that Endostar inhibits angiogenesis and that the downregulation of the Wnt/β-catenin signaling pathway may be involved in the inhibition of angiogenesis by Endostar. These results support the use of Endostar in further clinical applications.
